# Berberine and magnolol exert cooperative effects on ulcerative colitis in mice by self-assembling into carrier-free nanostructures

**DOI:** 10.1186/s12951-024-02804-x

**Published:** 2024-09-04

**Authors:** Yida Xu, Zhejie Chen, Wei Hao, Zhengming Yang, Mohamed Farag, Chi Teng Vong, Yitao Wang, Shengpeng Wang

**Affiliations:** 1 State Key Laboratory of Quality Research in Chinese Medicine, Institute of Chinese Medical Sciences, University of Macau, Macau, China; 2 Macao Centre for Research and Development in Chinese Medicine, Institute of Chinese Medical Sciences, University of Macau, Macau, China; 3 Institute of Molecular Medicine (IMM), Shanghai Key Laboratory for Nucleic Acid Chemistry and Nanomedicine, Renji Hospital, School of Medicine, Shanghai Jiao Tong University, Shanghai, 200127 China; 4 Pharmacognosy Department, College of Pharmacy, Cairo University, Kasrel Aini St., Cairo, 11562 Egypt

**Keywords:** Berberine, Magnolol, Ulcerative colitis, Self-assembly, Gut microbiota

## Abstract

The risk of ulcerative colitis (UC) is increasing worldwide with limited success using classical drugs, which has underscored the development of novel agents. Recently, carrier-free molecular assembly has been proven to be an effective drug delivery system, but it has yet to be examined for UC drug development using phytochemicals. Based on traditional Chinese medicine compatibility and potential medicinal uses, a pair of natural compounds, berberine (BBR) and magnolol (MAG), were found to self-assemble into nanostructures in aqueous solutions. Spectral analysis revealed that the assembly mechanisms of BBR and MAG were mediated through charge interactions and *π-π* stacking. Pharmacokinetic studies and animal imaging showed that BBR-MAG self-assembly (BM) effectively promoted the oral bioavailability and biodistribution of BBR in the colon. BM exhibited superior effects in regulating inflammatory factors, maintaining colon barrier integrity, and regulating gut microbiota in a dextran sulfate sodium salt-induced colitis mouse model. Additionally, no apparent signs of toxicity were observed, suggesting that BM has a favorable safety profile. This study presents a new strategy for UC management and highlights the cooperative effects of combined phytochemicals.

**Graphical Abstract:**

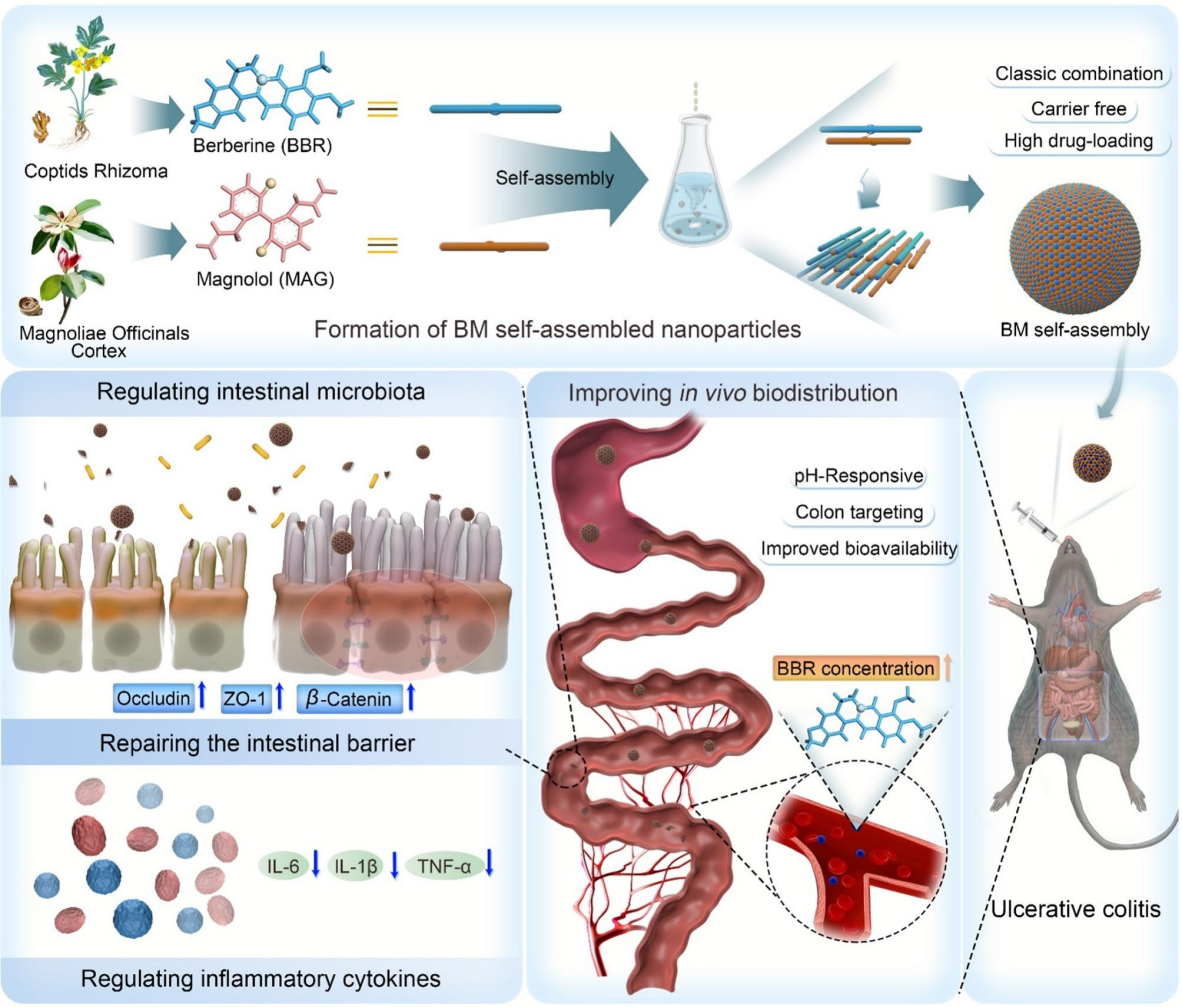

**Supplementary Information:**

The online version contains supplementary material available at 10.1186/s12951-024-02804-x.

## Introduction

In recent years, the discovery and design of self-assembling organic molecule pairs have become a rapidly developing field. Compounds with specific chemical structural properties have been self-assembled into nanoparticles to enhance antibacterial effects [1–3], reduce toxicity [4], improve selectivity [5], and enhance therapeutic efficacy [6]. The use of natural small molecule self-assembled systems offers several advantages, including high drug loading rates, good biocompatibility, controllable degradability, and pharmacological activity with synergistic effects [7]. So far, research on self-assembly has mainly focus on physicochemical properties and in vitro effects, while research on formation mechanisms and in vivo process is relatively limited.

Ulcerative colitis (UC) is a chronic condition that affects the colon and is classified as one of the inflammatory bowel diseases (IBD). The development of UC involves a combination of genetic predisposition, epithelial barrier defects, dysregulated immune responses, and environmental factors [8]. The occurrence and prevalence of UC are rising globally, making it a widespread condition [9]. Numerous studies have demonstrated that UC is a costly disease, with medical expenses estimated to be three to four times higher than the population average income [10]. The use of antibiotics, antitumor necrosis factor (anti-TNF-*α*) antibodies, amino salicylates, corticosteroids, and thiopurines are the traditional approaches for UC management. Long-term use of these medications, however, can cause side effects such as diarrhea, headache, nausea, osteoporosis, and vomiting, and may diminish their effectiveness. Approximately one-third of UC patients require surgical intervention after long-term reliance on these conventional treatments. Scientific evidence indicates that herbal medicines and natural products, such as baicalein, indigo naturalis, andrographolide, curcumin, and bromelain, have demonstrated efficacy in the treatment of UC [11, 12].

Huanglian-Houpo Decoction, composed of Coptidis Rhizoma and Magnolia Officinalis Bark, is a renowned classical prescription of traditional Chinese medicine that has been frequently used to treat gastrointestinal tract diseases in the clinic since the Ming dynasty (AD 1390). The mechanism of Huanglian-Houpo Decoction in treating UC and its efficacy have been reported in both TNBS (2,4,6-Trinitrobenzenesulfonic acid)-induced rat UC model and dextran sulfate sodium (DSS)-induced mouse UC model [13, 14]. Berberine (BBR), a representative active compound of Coptidis Rhizoma, has been shown to possess promising therapeutic effect for the treatment of UC [15]. Magnolol (MAG), a representative lignan derived from Magnolia Officinalis Bark, has also been reported to possess potential anti-inflammatory activity [16, 17].

Inspired by the compatibility of traditional Chinese medicine and current research, we conducted this research on BBR and MAG. Based on extensive *in silico* computation and in vitro characterization, we discovered that BBR and MAG could self-assemble into stable nanoscale structures in aqueous solution. We then elucidated the self-assembly mechanism and identified the conformation of the complex unit. In vivo assays on rat and colitis mice demonstrated that these nanosized-assembled particles exhibit enhanced absorption, targeting ability, and therapeutic efficacy against UC. These results highlight the cooperative effect of combined phytochemicals for the management of UC.

## Materials and methods

### Chemicals and reagents

Berberine (98% purity) was purchased from Shanghai Yuanye Biological Technology Co., Ltd. (Shanghai, China), and magnolol (98% purity) was purchased from Baoji Herbest Bio-Tech Co., Ltd. (Baoji, China). Berberine-d6 (95% purity), as internal standard for mass spectrometry analysis, was provided by CATO Research Chemicals Inc (Guangzhou, China). MCE China (Shanghai, China) produced the 1,1-dioctadecyl-3,3,3,3-tetramethylindotricarbocyaine iodide (DiR, 99% purity). The enzyme-linked immunosorbent assay (ELISA) kits were provided by BioLegend, Inc. (San Diego, CA, USA), including ELISA MAX™ Deluxe Set Mouse IL-1*β* (432615), IL-6 (31315), TNF-*α* (430207), and IFN-*γ* (430815). The antibodies for immunofluorescence were provided by Proteintech Group, Inc (Wuhan, China), including BrdU monoclonal antibody (1B10E12,66241-1-Ig), *β*-Catenin polyclonal antibody (51067-2-AP), ZO-1 polyclonal antibody (21773-1-AP), and Occludin polyclonal antibody (13409-1-AP). Merck & Co Inc. (Rahway, NJ, USA) provided the 5-Aminosalicylic acid (5-ASA) (95% purity), and MP Biomedicals LLC (Irvine, CA, USA) provided the DSS (Mw 36–50 kDa). Other reagents were analytical grade.

### Preparation of BM

**Fig. 1:**
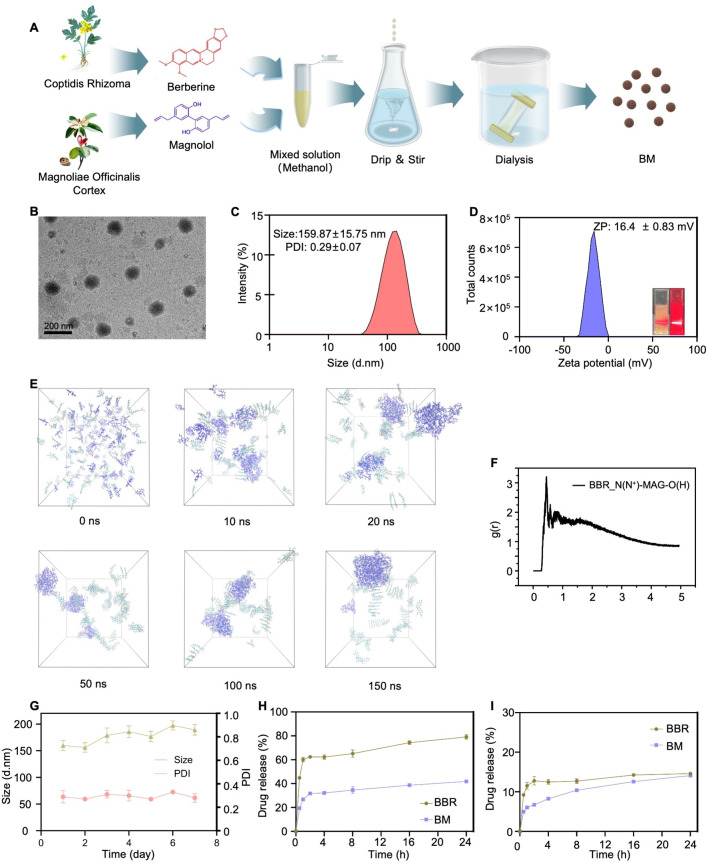
BBR and MAG formed stable nanoassembly in aqueous solution: (**A**) Preparation process of BM; (**B**) BM morphology under transmission electron microscopy (TEM) (**C**) Size distribution of BM; (**D**) Zeta potential of BM and Tindall effect of BM solution; (**E**) Fully atomistic simulation of BM at different simulation time (0–150 ns); (**F**) Radial distribution functions of BM; (**G**) DLS results regarding the size and PDI of BM for seven days; (**H**) Drug release of BM and BBR in weakly alkaline (pH 7.4) and (**I**) acidic solutions (pH 2.4)

To prepare the BBR and MAG nano-assemblies, both compounds were dissolved in methanol at a 1: 1 molar ratio to create a 10 mmol/mL stock solution. An ultrasonic instrument (CR-009 S, Chunlin, China) was employed to assist in the dissolution process. Subsequently, 100 *µ*L of the stock solution was added dropwise to 1 mL of water while stirring. The resulting mixed solution was then heated to 70℃ and stirred for 30 min to evaporate the methanol. The reaction system was dialyzed in water to remove free BBR and MAG, yielding the self-assembled BM (Fig. 1A). The BM nano-assembly solution was then lyophilized using a MODULYOD-230 lyophilizer (ThermoFisher Scientific, Waltham, MA, USA) and stored at 4℃ for further use.

### Characterization of BM

Dynamic Light Scattering (DLS) instrument (Zetasizer Nano ZS, Malvern Panalytical, Malvern, UK) was utilized to determine the zeta potential, polydispersity index, and particle size of BM. The measurements were performed in water at 25 °C using disposable cuvettes (ZEN0040). Over the course of one week, BM samples were stored at room temperature (25 °C) and measured the particle size daily. Transmission electron microscopy (TEM; JEM-1400, JEOL, Tokyo, Japan) was employed to capture morphological images of the BM samples, with a point resolution of 0.25 nm and a line resolution of 0.102 nm. Furthermore, X-ray diffraction (XRD) spectra (D8 ADVANCE, Bruker, Karlsruhe, Germany) of free BBR, BM, and MAG was obtained using a scanning set at 40 kV and 40 mA from 5° to 80°. The step size was 0.02°, and copper was used as the target material. Infrared spectra were obtained by compressing the sample with potassium bromide (FT-IR grade, Sigma, China) at a 1:100 ratio, and measured using a Fourier-transform infrared (FT-IR) spectrophotometer (Nicolet iS10, Thermo Fisher Scientific). Data collection was performed 200 times with a step size of 4 cm⁻¹ from 4000 cm^−1^ to 500 cm^-1^. The ^1^H nuclear magnetic resonance (NMR) and ROESY two-dimensional (2D) NMR (Bruker-600 MHz, Bruker) spectra were obtained and the Mestrenova software (Mestrenova, Mestrelab, Escondido, CA, USA) was used for the analysis.

### Molecular dynamics simulation of BM

The Packmol software (State University of Campinas, São Paulo, Brazil) was used to construct two types of molecular mixtures at a 1: 1 ratio, with 100 molecules of each type. The constructed mixed model was then placed in a pre-equilibrated TIP3P aqueous phase containing 8000 water molecules, within a box size of 100 × 100 × 100 Å³. Chloride ions were added to neutralize the charge of the entire system. The cutoff distance for non-bonding interactions was set to 12 Å, and the PME method was used to handle long-range electrostatic interactions. The OPLS-AA force field was employed to describe the entire simulation system. A 0.1 ns V-rescale thermostat and a 0.1 ns Parrinello-Rahman constant pressure regulator were used to achieve preliminary equilibrium of the water solvent distribution. Finally, a 200 ns molecular dynamics (MD) relaxation was conducted with an integration time step of 2 fs, under a simulated temperature of 298.15 K and atmospheric pressure of 1 atm. The LINCS algorithm was used to constrain all covalent bonds involving hydrogen atoms. The molecular dynamics simulation process was completed using the GROMACS software package (University of Groningen, Netherlands), and visualizations were created using VMD 1.9.1 (University of Illinois Urbana-Champaign, USA).

### In vitro release assay of BM

To measure the in vitro cumulative release of BM, the sample was gently agitated in PBS buffer (pH 7.4) at 37 °C with a magnetic stirrer. Specifically, 2 mL of 2.5 *µ*mol/mL BM and BBR were placed in a dialysis bags (MWCO 3000 Da, Sigma-Aldrich, St. Louis, MO, USA). The bags were then immersed in an acetate buffer (pH 2.0) and 20 mL of PBS (pH 7.4) at 37 °C, stirred at 200 rpm [18]. At intervals of 0.5, 1, 2, 4, 8, 16, and 24 h, 500 *µ*L aliquots of the solution was collected, replacing each by an equal volume of fresh PBS solution. High-performance liquid chromatography (HPLC; Waters 2998, Waters Corp., Milford, MA, USA) was conducted to determine the concentration of BBR at a wavelength of 365 nm. The isocratic mobile phase consisted of A: methanol and B: 0.1% formic acid in water (30% A/70% B, v/v). The flow rate was 0.3 mL/min. To assess accuracy, the experiment was conducted in triplicate.

### Pharmacokinetic analysis

The female and male Sprague-Dawley (SD) rats was categorized into BM and BBR groups. The BBR and BM were administered orally at 0 h (converted the same dosage to BM at 55 mg/kg). At 13 time points (0.08, 0.16, 0.25, 0.33, 0.5, 0.75, 1, 2, 3, 5, 8, and 24 h), blood samples (250 to 300 *µ*L) were collected from the orbit vein. The blood samples were collected in an EP tube containing ethylenediaminetetraacetic acid dipotassium salt dihydrate (ED-K2) and centrifuged at 3000 rpm at 4℃ for 10 min. For subsequent analysis, the plasma was stored the at -80 °C. Additionally, BM and BBR were orally administered, and the rates were sacrificed after administering the dose for 3 h. Then the entire colon tissues were collected, cut into about 2 mm pieces and mixed with a radioimmunoprecipitation assay (RIPA) lysis solution (Beyotime, Beijing, China) at the ratio of tissue weight (g): lysis solution volume (mL) = 1: 2 in the presence of magnetic beads. The mixture was homogenized for 10 min and stored at -80 °C for further analysis.

Then, BBR standard substance was dissolved in 50% methanol to prepare standard working solutions with concentrations of 10, 20, 50, 250, 1000, 2500, 5000, and 10,000 ng/mL. 5 *µ*L of this solution was combined with 45 *µ*L of blank plasma to create standard samples with concentrations of 1, 2, 5, 25, 100, 250, 500, and 1000 ng/mL. Quality control (QC) standards were prepared at concentrations of 2.5, 100, and 800 ng/mL. Berberine-d6 was dissolved in 50% methanol at a concentration of 5000 ng/mL for use as the internal standard. 5 *µ*L of the internal standard was combined with a 50 *µ*L sample of rat plasma or colon homogenate, along with 0.2 mL of methanol, and vortexed for 3 min. Subsequently, the mixture was centrifuged in a high-speed frozen centrifuge at 14,000 rpm/min for 10 min at 4 °C (543R, Eppendorf, China). Finally, 0.15 mL of the supernatant was carefully pipetted into the inner lining tube for sample analysis. The plasma and colon homogenate were analyzed by liquid chromatography-mass spectrometry (LC-MS; Triple Quad 4000, AB Sciex, Framingham, MA, USA), MS conditions are represented in supplementary material Table S1 and S2. The representative chromatogram and mass spectrum of samples are shown in Figs. S3 and S4.

### In vivo distribution and retention studies

DiR were loaded to BM to conduct an in vivo distribution and retention study [19]. To obtain DiR@BM, 500 *µ*g/mL DiR was dissolved with BBR and MAG (10 mmol/mL) in method as working solution. Then the 100 *µ*L working solution was added dropwise to the 1 mL dd-water (Double-distilled water) while string (200 rpm). The resulting mixed solution was heated at 70 °C and stirred to evaporate the methanol for 30 min. The obtained supernatant was centrifuged at 4500 rpm and then ultrafiltration for 10 min to precipitate insoluble and free DiR. 6 male C57BL/6 mice were randomly divided into DiR@BM and free DiR groups. DiR@BM and DiR were orally administered to each group of mice (convert the same dose to BBR at 25 mg/kg). After oral administration, an in vivo imaging system (Lumina XR III, USA) was used to scan the animals in the different groups at 6, 12, and 18 h. The imaging mode is florescent with an excitation wavelength at 760 nm and an emission wavelength at 790 nm. The mice were sacrificed after the last time point and then the colons were scaned.

### Therapeutic efficacy of BM in DSS-induced acute colitis mice

Male C57BL/6 mice (8 to 10 weeks) was randomly assigned into seven groups (*n* = 6), including control, DSS, 5-ASA, BBR, MAG, BBR-MAG, and BM groups. We housed and acclimatized the mice for seven days before the experiment. To trigger experimental colitis, we gave the mice 3% DSS in drinking water for 10 days. 5-ASA (100 mg/kg), BBR, BBR-MAG, and BM (the dosage calculated as BBR and MAG was 25 and 16 mg/kg, respectively) were orally administered to the animals in 5-ASA, BBR, MAG, BBR-MAG, and BM groups, respectively, on days 3, 5, 7, and 9. The weight changes and the disease activity index (DAI) of the mice were recorded every day. On day 10, the mice were sacrificed, and the colons were collected. Hearts, livers, spleens, lungs, and kidneys of mice in each group were also collected to evaluate the toxicity of BM. For the microbiome analysis, the feces were collected and stored at -80℃.

The ELISA kits were used to detect the expression levels of the cytokines (Il-1*β*, IL-6, IFN-γ and TNF-α) in the colon tissues. The immunofluorescence (IF) assay was used to assess the positive effect of BM on intestinal barrier protection as well as levels of β-Catenin, ZO-1 and occludin. The BrdU monoclonal antibody was used to stain the sections of the colon and examine the protective effect of BM on intestinal cell apoptosis. A Nikon Eclipse Ts2R-FL microscope system (Nikon, Tokyo, Japan) was used to observe the stained sections. ImageJ software (ImageJ, National Institutes of Health, Bethesda, MD, USA) was used for the quantitative determination of images.

### Microbiota analysis

On day 10, the mouse fecal samples were collected and moved for microbiome analysis to Majorbio Bio-Pharm Technology Co., Ltd., on dry ice. The E.Z.N.A.^®^ soil DNA Kit was used to extract the DNA, which we separated on agarose gel. DNA concentration and purity were determined by applying a NanoDrop 2000 UV-vis spectrophotometer. Prim-r pairs 338 F and 806R and an ABI GeneAmp^®^ 9700 PCR thermocycler (GeneAmp, Thermo Fisher) were used to amplify the hypervariable region V3-V4 of the bacterial 16 S rRNA gene. The Illumina MiSeq sequencing platform with PE300 chemical was used to sequence the purified amplicons. The Majorbio Cloud Platform (www.majorbio.com) was used to perform a related analysis of the 16 S rRNA microbiome sequencing data.

### Statistical analyses

Data analyses were performed using the one-way analysis of variance (ANOVA) and nonparametric Tukey’s test. *p < 0.05, **p < 0.01, and ***p < 0.001 were indicated that the difference between the groups was statistically significant.

## Results

### BBR and MAG can form stable nanoassembly in aqueous solution

BM was formed through self-assembly from nature products BBR and MAG in aqueous solutions (Fig. S1). A clear Tyndall light-scattering effect was observed in the BM solution (Fig. 1D). According to the DLS analysis, the average particle size of BM was 159.87 ± 15.75 nm, and the polydispersity index (PDI = Mw/Mn) was 0.29 ± 0.07 (Fig. 1C). Additionally, the surface charge of BM solution was measured to be 16.4 ± 0.83 mV (Fig. 1D). The topography of BM transmission electron microscope was shown in Fig. 1B. TEM and DLS results were mutually confirming in the size of the BM. A MD simulation was employed to visualize MAG interactions and assess the stability of the self-assembled nanostructures. These results demonstrated a spontaneous aggregation process of the BM system (Fig. 1E). Our initial observation revealed that BBR and MAG was randomly distributed in the continuous phase, followed by the formation of the first aggregate. Finally, BM was found to be tightly interlinked, forming a spherical structure within 150 ns. Notably, MAG molecules were securely embedded within BBR molecules through hydrogen and hydrophobic interactions. The radial distribution function was used to characterize the aggregation tendency of nitrogen atom in BBR and oxygen atom in MAG (Fig. 1F). We also observed a mass spectrum peak at m/z = 602.24, which was equal to the sum of the molecule weights of berberine and magnolol. This result also verified the formation of BM aggregates (Fig. S2).

According to the DLS results, particle size and PDI did not change significantly over a duration of seven days (Fig. 1G), which indicated that BM had good stability. In vitro release showed that at pH 7.4, the release rate of BM was lower than that of free BBR (Fig. 1H). At pH 2.4, free BBR basically reached equilibrium within 4 h, whereas the release of BM continued until 20 h (Fig. 1I). These results suggest that BM nano-assembly was relatively stable and released less in the stomach, which allowed it to reach the colon to exert its intended therapeutic effect.

### BM is formed by electrostatic attraction and π–π stacking

**Fig. 2:**
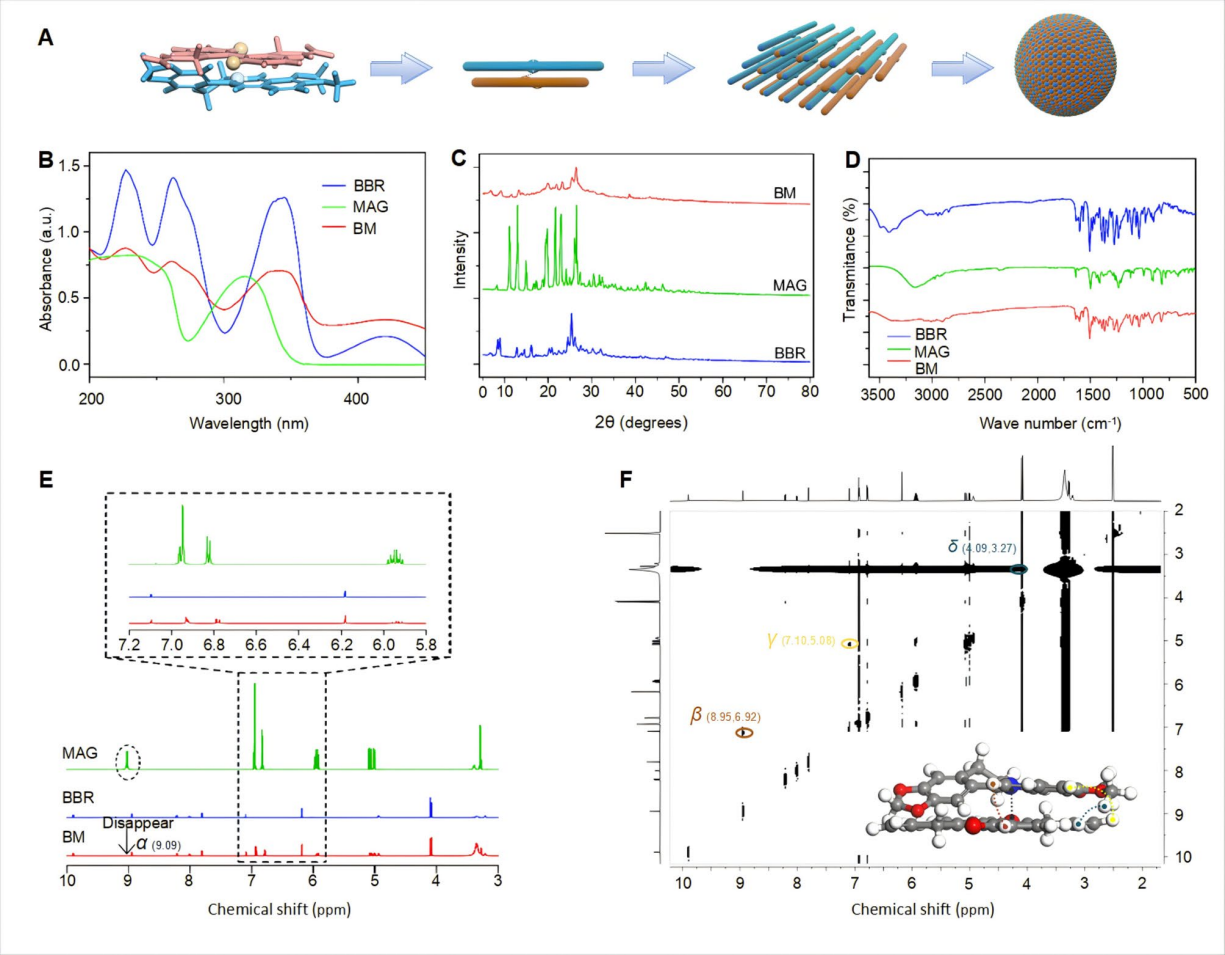
BM was formed by electrostatic attraction and *π–π* stacking. (**A**) Self-assembling mode between BBR and MAG in BM; (**B**) UV-vis spectra of BBR, MAG, and BM; (**C**) XRD spectra of BBR, MAG, and BM; (**D**) FT-IR spectra of BBR, MAG, and BM; (**E**) ^1^H-NMR spectra of BBR, MAG, and BM; (**F**) ROESY 2D NMR spectrum correlations of BM and signal annotation in spatial structure

Figure 2B showed the UV absorption spectrum. The BBR UV absorption peaks were at 227, 262, and 345 nm, and the MAG absorption peak was at 236 nm. The BM UV absorptions reached the maximum at 226, 261, and 343 nm, which demonstrated the simultaneous presence of MAG and BBR in BM. The widened and weakened UV peak suggested the formation of larger size molecules in the solution. MAG had strong and sharp diffraction peaks at 10–30°, according to the XRD analysis, which revealed its crystal structure. The four highest intensity peaks at 8.98°, 16.12°, 25.36°, and 26.06° revealed a series of obvious crystal diffraction peaks. BM, however, did not have corresponding characteristic peaks, which revealed that BM was amorphous after assembly. Because the vibration mode among atoms did not demonstrate Raman activity, crystal diffraction peaks were lacking (Fig. 2C).

To elucidate the underlying mechanism of BM self-assembly, FT-IR was applied to reveal changes in the molecular vibrations of the constituent compounds. After assembly, a weakening of the band at 3157 cm^-1^ was observed, which resulted in a wide peak, showing that phenolic groups formed inter-molecular associations during self-assembly. At the higher wavenumber of 1604 cm^-1^, a band at 1598 cm^-1^ was observed that correlated with C = N stretching vibration from BBR. We attributed this shift to an increase in the electron cloud density of the conjugated system involving nitrogen cations after assembly, resulting in an increase in C = N bond energy. In contrast, the band at 1638 cm^-1^ corresponding to the C = C stretching vibration from MAG shifted to a lower wavenumber of 1635 cm^-1^, which suggested a decrease in electron absorption after the ionization of protons from phenolic hydroxyl groups. This result indicated a weakening of the polarity and energy of the C = C bond (Fig. 2D).

To confirm the constitution of the nanoassembly, a ^1^H NMR was conducted. As shown in Fig. 2E, the single peak at a chemical shift of 9.09 ppm (signal *α*) disappeared, which proved the ionization of phenolic hydroxyl groups in MAG during the formation of BM, consistent with the FT-IR results. Moreover, the peaks at chemical shifts of 6.95 and 6.82 ppm shifted toward 6.91 and 6.76, respectively, which suggested potential *π–π* stacking interactions between aromatic rings. The ROESY spectrum was also conducted to provide insight into the binding conformation of molecules in BM in Fig. 2F. Signals *β*, *γ*, and *δ* represented the strong interaction between H-10´, H-10´, and H-23/25´ from BBR and H-9/10´, H-17/20´, and H-15/18´ from MAG, respectively [2]. We drew the four signals in three-dimensional (3D) molecular pairs in corresponding color (Fig. 2F), and the result was consistent with the spatial conformation. In summary, the ionized hydroxyl groups of MAG and the nitrogen cations of BBR generated electrostatic interactions, leading to the aggregation of molecules in the solution and the formation of a stable nano-assembly, as depicted in the schematic (Fig. 2A).

.

### BM improves the bioavailability and colon accumulation compared with free BBR

**Fig. 3:**
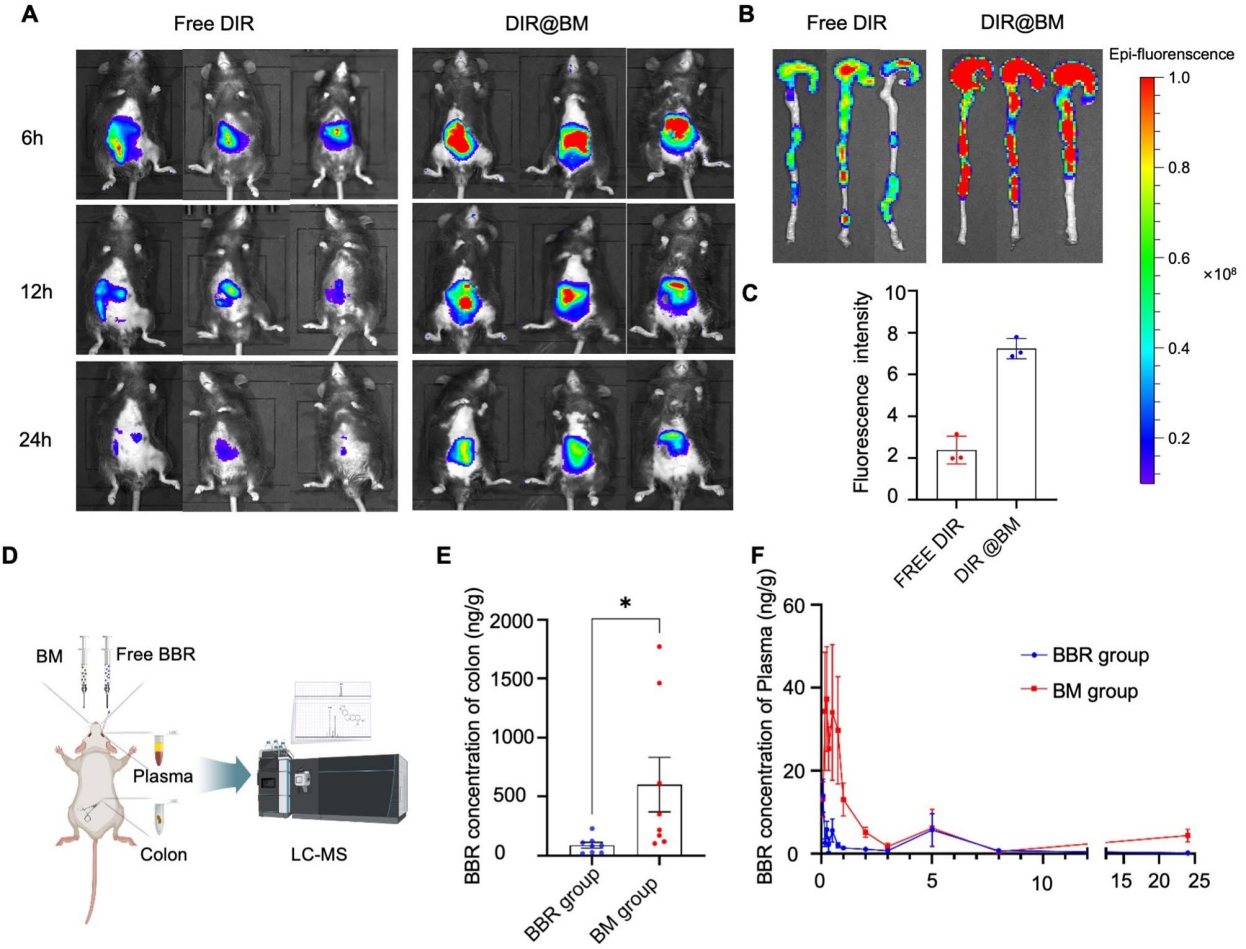
BM improved the bioavailability and colon accumulation compared with free BBR: (**A**) In vivo fluorescence images of oral deliverance of DiR@BM to mice; (**B**) Fluorescence images of mice colon isolated after 24 h drug administration; (**C**) Living Image 4.5 software measurements of the region of interest (ROI) fluorescence intensity of colons (*n* = 3); (**D**) Pharmacokinetic analysis process of BM and free BBR in SD rats; (**E**) BBR concentration of BBR in colon tissue after administration for 3 h (*n* = 7); (**F**) BBR concentration in plasma (*n* = 7). Significance is denoted as *p < 0.05, **p < 0.01, and ***p < 0.001; values are denoted by mean ± SD

To track the targeted effectiveness of BM in UC mice, DiR was loaded into the BM system to obtain BM@DiR. The BM@DiR solution and free DiR was administered orally to male C57BL/6 mice. At 6, 12, and 18 h after administration, the average fluorescence intensity of the free DiR group was lower than that of the BM@DiR group according to the results of the in vivo imaging system (IVIS; Fig. 3A). The average fluorescence intensity of the colon in the free DiR group was lower than that in the BM@DiR group (Fig. 3B and C). Thus, after oral administration, colonic distribution of BBR was promoted by the BM nanostructure assembly.

To further verify the in vivo distribution of BM, the pharmacokinetic studies were conducted on SD rats. The mean pharmacokinetic curves were presented in Fig. 3F. The maximum serum level (C_max_) of BM (63.7 ± 39.4 ng/mL) was significantly higher than that of BBR (16.8 ± 15.5 ng/mL, *p* = 0.0126). Meanwhile, the area under the curve (AUC_0-t_) of BM (96.255 ± 74.895 ng/L × h) was 3.24 times higher than that of BBR (29.85 ± 21.752 ng/L × h, *p* = 0.0421), which we attributed to the solubilization effect formed by the nanoassembly that improved oral bioavailability [20, 21]. The BBR level in the colon tissue 3 h after oral administration Was also determined. As shown in Fig. 3E, the tissue level of BBR in the BM group was significantly higher than that in the free BBR group. We speculate that BM was more readily endocytosed by intestinal epithelial cells than by free BBR. Other studies have shown that MAG can serve as a potential inhibitor of P-glycoprotein [22, 23], thereby reducing the exocytosis of BBR.

### BM shows therapeutic efficacy advantages against DSS-induced colitis

**Fig. 4:**
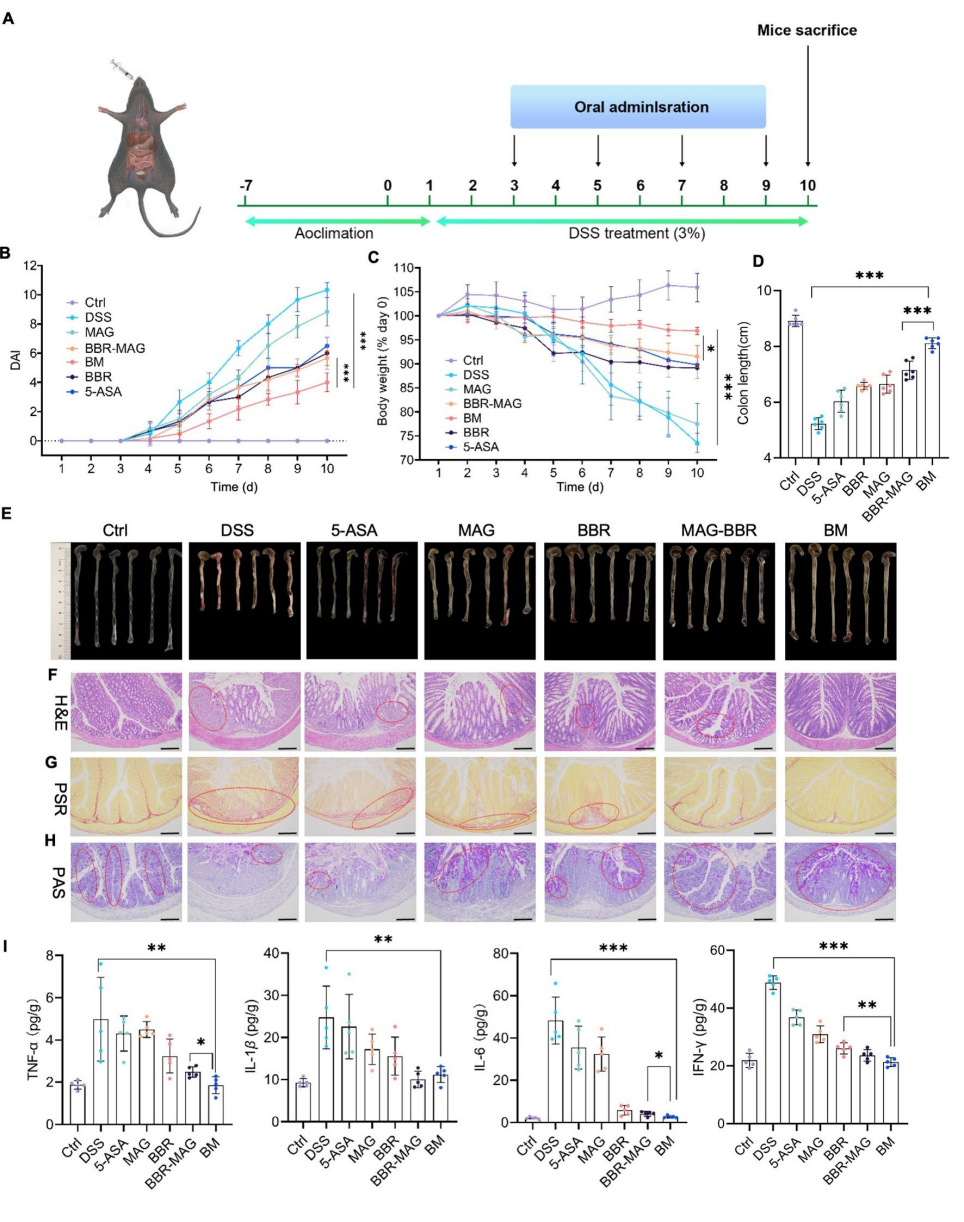
BM showed therapeutic efficacy advantages against DSS-induced colitis: (**A**) Drug intervention plan; (**B**) Body-weight changing trend, (**C**) DAI changing trend, and (**D**) Colon length of various groups (*n* = 6); (**E**) Digital colon photos isolated from mice; Representative images of (**F**) H&E-stained histological section (scale bar: 100 *µ*m), (**G**) PAS-stained histological section scans, and (**H**) PSR-stained histological sections (scale bar: 200 *µ*m); (**I**) TNF-*α*, IL-6, IL-1*β* and INF-*γ* levels in colon tissues isolated from various groups (*n* = 5). Significance is denoted as *p < 0.05, **p < 0.01, and ***p < 0.001; values are denoted as mean ± SD

The pharmacodynamics of BM was evaluated in mouse model of UC induced by DSS. The positive control drug, 5-ASA, commonly used as a first-line treatment for UC, was also used in the experiment for comparison of efficacy level. The mice were categorized into the following seven groups: Ctrl, DSS, 5-ASA, MAG, BBR, MAG-BBR, and BM groups. To establish the UC model, 3% DSS aqueous solution was used to replace drinking water for mice to the 5-ASA, BBR, BBR-MAG, BM, DSS, and MAG groups. From days 3 to 9, different intervention preparations administered to the mice orally every two days (Fig. 4A). Compared with the BM group, DSS aggravated body-weight loss and upregulated the DAI. The therapeutic effect of BM preparation was significantly better than that of the BBR-MAG combined administration group (Fig. 4B and C). Colon atrophy symptoms in UC were notably alleviated by BM. The colon lengths of the 5-ASA, DSS, and BBR-MAG groups were significantly shorter than that of the BM group (Fig. 4D and E).

According to hematoxylin and eosin (H&E) staining, local mucosal necrosis, abnormal intestinal morphology, branching of crypts in UC mice, and infiltration of inflammatory cells were evident in the stained sections. After BM treatment, these changes were significantly relieved, and their morphology was similar to that of the control group (Fig. 4F). UC is a complex disease that ultimately leads to extensive tissue fibrosis [24]. The results of Picrosirius red stained (PSR) sections showed that the fibrosis in the DSS group was most significant. MAG and BBR showed partial therapeutic effects on fibrosis, whereas the BM group did not show significant fibrotic symptoms (Fig. 4G). Goblet cells contributed properties essential for gastrointestinal homeostasis [25]. Periodic acid-Schiff stain (PAS) was used to visualize neutral mucus material in colonic goblet cells. The DSS group showed severe goblet cell apoptosis in the PAS assay results. Superior protective activity from BM was most effective on goblet cells (Fig. 4H). ELISA was used to detect inflammation levels in various groups of colon homogenates. Important inflammatory factors [26], such as TNF-*α*, IL-1*β*, IL-6, and INF-*γ* in the DSS group were significantly higher than those in the BM group. The result revealed that the self-assembled body had an anti-inflammatory effect. Additionally, the BBR-MAG group’s IL-6, TNF-*α*, and INF-*γ* levels were significantly higher than those of the BM group, which showed that the efficacy of the formulation had improved (Fig. 4I). Note that the reduction level in all levels of inflammatory mediators was stronger in the BM group than in the 5-ASA group.

### BM is protective of the epithelial barrier and limits cell apoptosis

**Fig. 5:**
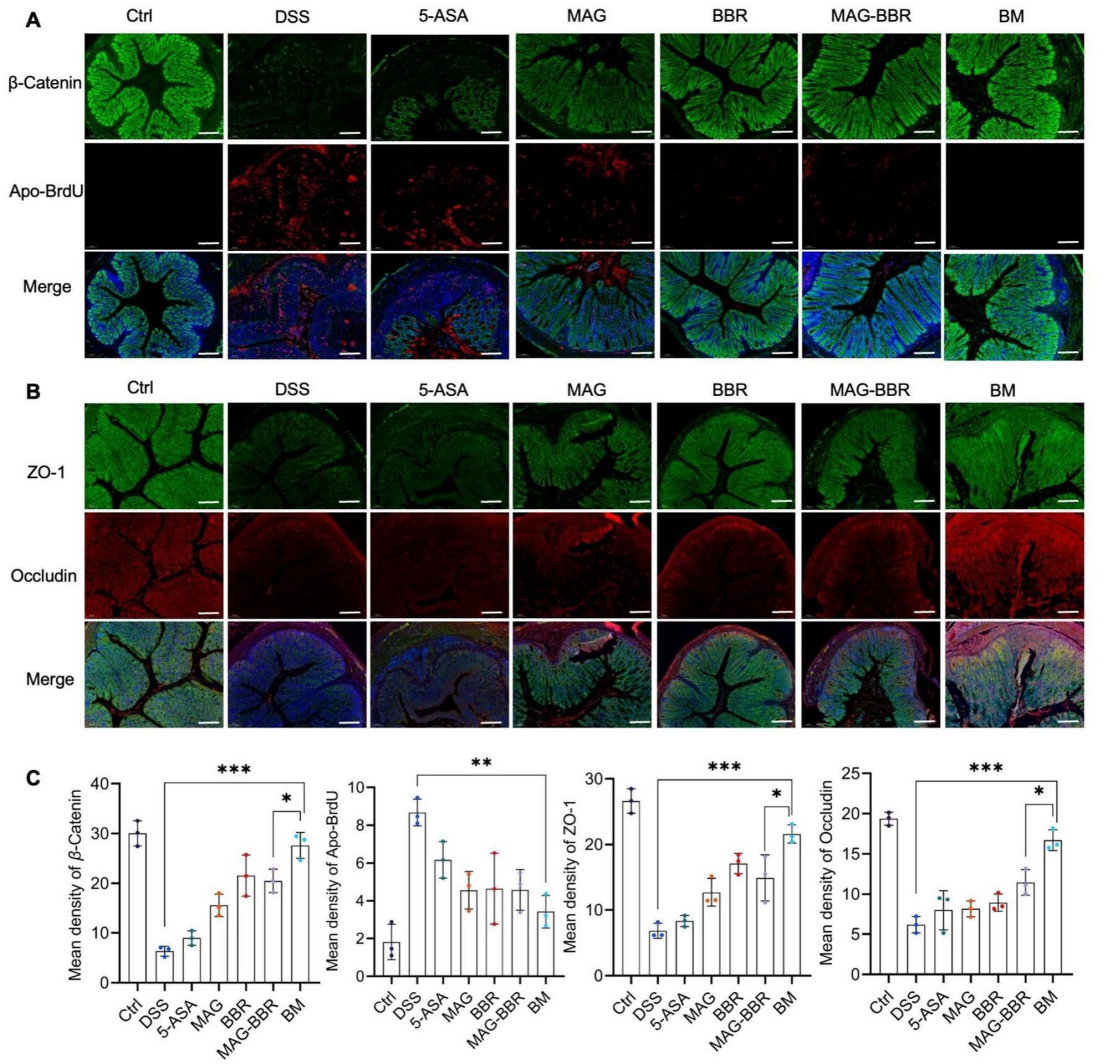
BM protected the epithelial barrier and reduces cell apoptosis: (**A**) IF images of *β*-catenin and apo-BrdU in colon tissues (scale bar = 100 *µ*m); (**B**) IF images of ZO-1 and occludin in colon tissues (scale bar = 100 *µ*m); (**C**) Histogram of semiquantitative analysis of *β*-catenin, ZO-1, apo-BrdU, and occludin in mouse colons. Significance is denoted as *p < 0.05, **p < 0.01, and ***p < 0.001; values are denoted as mean ± SD (*n* = 3)

The foundation of intestinal health is an intact epithelial barrier. In the UC pathological conditions, these tight junction proteins were disrupted [27–29]. To determine the expression levels in the colon of *β*-Catenin, ZO-1, and occludin, IF was conducted. A significant downregulated ZO-1 expression was observed in the DSS group compared with the control group. The therapeutic effects of 5-ASA, MAG, BBR, and MAG-BBR increased sequentially, whereas the therapeutic effects of BM were significantly better than the previously noted treatment groups (Fig. 5A and B). Meanwhile, a semiquantitative determination of *β*-Catenin, ZO-1, and occludin confirmed these results (Fig. 5C). Apoptosis is considered to be one of the main causes of epithelial dysfunction in UC [30]. IF was used to detect the expression of Apo-BrdU in colon tissue. The image results and semiquantitative assays confirmed that the BM treatment significantly reduced Apo-BrdU in the UC intestine.

### BM regulates intestinal microbiota in DSS-induced colitis

**Fig. 6:**
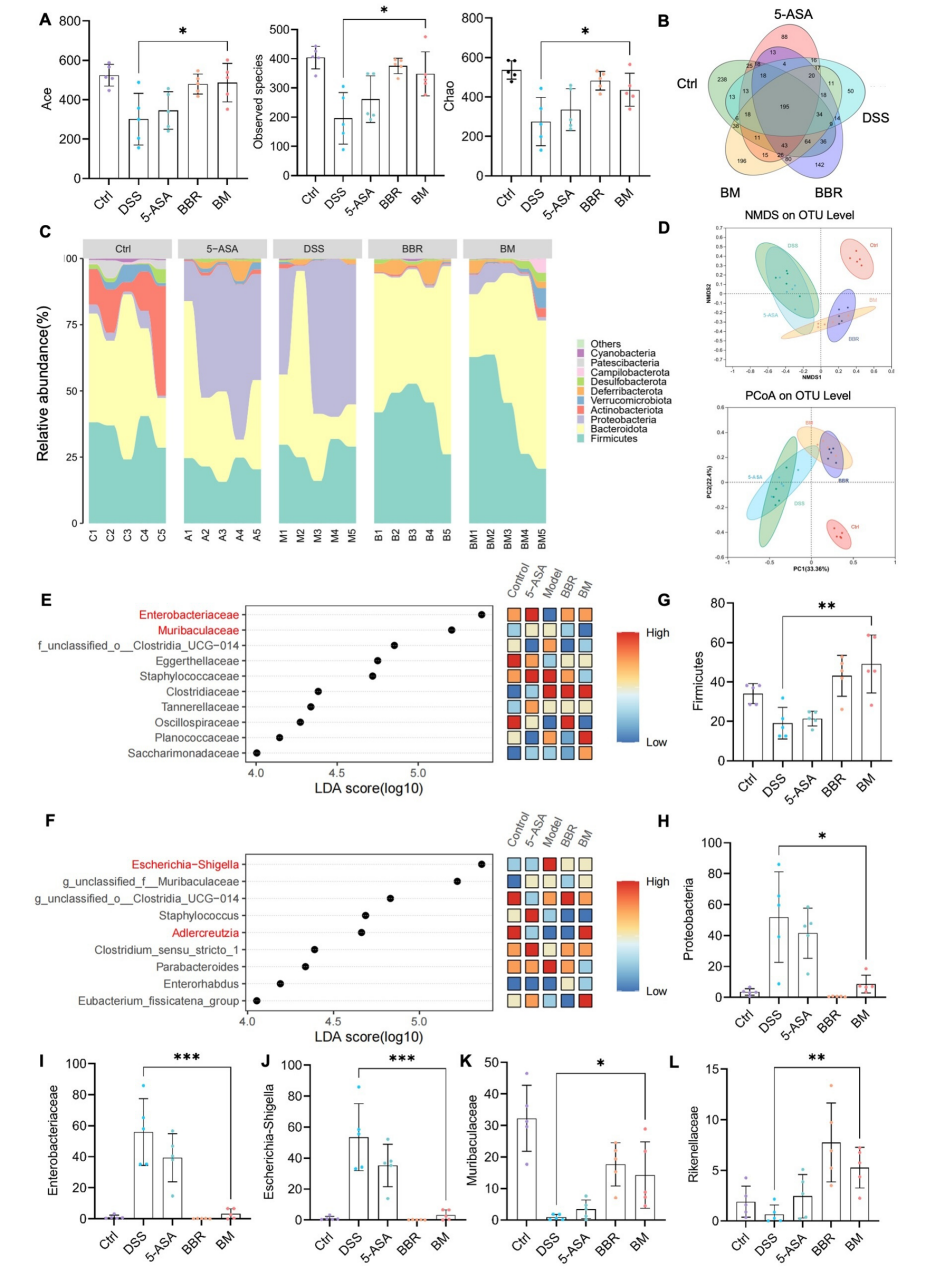
Intestinal microbiota regulated by BM in colitis induced by DSS: (**A**) Alpha diversity index for Ace, observed species, and Chao; (**B**) Venn diagram of five groups of species; (**C**) Area plot of cross groups showing phylum abundance; (**D**) NMDS and PCoA analysis of the level of operational taxonomic units; (**E**) Family-level LEfSe analysis with top 10 features according to significance; (**F**) Genus-level LEfSe analysis; (**G** and **H**) individual phylum abundance; (**I** and **L**) individual family and genus abundance. Significance level: *p < 0.05, **p < 0.01, and ***p < 0.001; values expressed as mean ± SD (*n* = 5)

Intestinal mucosal barrier integrity typically depends on how it interacts with gut microbiota. In UC pathogenesis, the commensal bacterial community has been implicated [31]. Therefore, 16 S rRNA gene amplicons in fecal samples were sequenced to assess gut microbiota composition. Based on the Observed species, Ace and Chao1, the α-diversity analysis showed that the disruption of species diversity was significantly increased by BM in the control model compared with the UC group (Fig. 6A). The Venn diagram also revealed that the BM group maintained the highest species diversity, second only to the control group (Fig. 6B). To evaluate the gut microbiota, the principal coordinates analysis (PCoA) was used. There is a significant dispersion phenomenon in the DSS group. After BM treatment, we observed a phenomenon of being segregated from the DSS group (Fig. 6D), which indicated that BM alleviated microbial dysbiosis in the UC model. Important UC features included increased Proteobacteria abundance and depleted anti-inflammatory bacteria (e.g., firmicutes) [32, 33]. The abundance stacking plot at the gate level showed a high similarity between the BM and control groups, with firmicutes significantly upregulated and proteobacteria significantly downregulated (Fig. 6C and G, and 6H).

To analyze the differences in specific bacteria among different treatment groups, we conducted linear discriminant analysis effect size (LEfSe) analysis at the species and genus levels and sorted results according to significance (Fig. 6E and F). The bacterial groups highlighted in red are species and genus with significant therapeutic effects on BM. At the species level, the scores of enterobacterea and multiaculaceae were the highest (LDA score > 5), which demonstrated the advantage of BM in regulating these two colon bacteria (Fig. 6I and J). At the genus level, BM had a significant effect on the regulation of Escherichia-Shigella (LDA score > 5) compared with the model group (Fig. 6K).

### BM shows good safety in mice

**Fig. 7:**
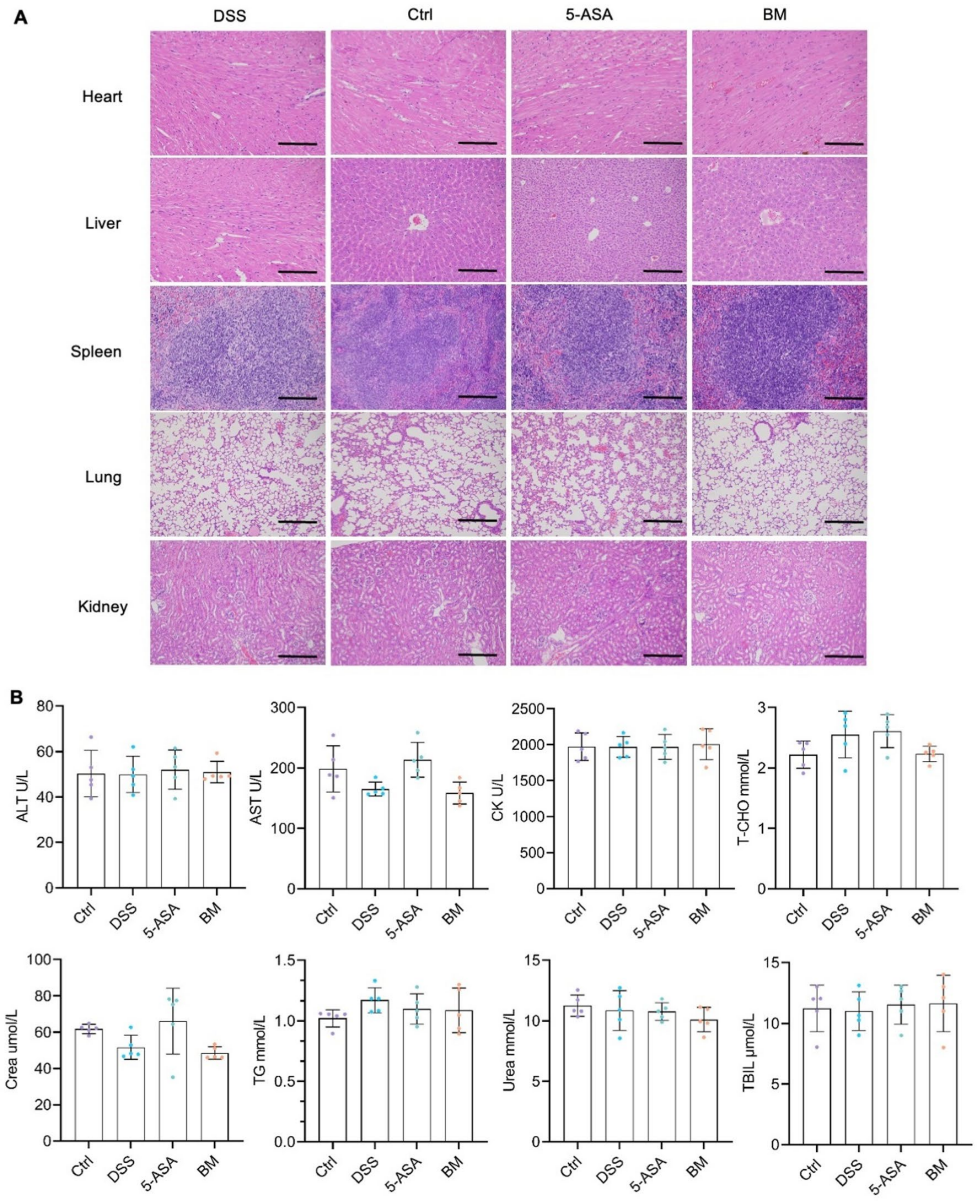
BM showed good safety in mice: (**A**) Scanning images of the mice heart, liver, spleen, lungs, and kidneys after H&E staining; (**B**) Individual blood biochemistry analysis. Scale bar = 100 *µ*m; values expressed as mean ± SD (*n* = 5)

BBR and MAG are the representative active compounds of traditional Huanglian Houpo decoction, which are commonly used in modern research for synergistic drug delivery [34, 35]. Due to the utilization of a carrier-free drug delivery system, the system only contains two natural compounds and some structural water, theoretically ensuringthe safety of this formulation. The heart, liver, spleen, lungs, and kidneys from 5-ASA, BM, DSS, and control groups were collected, and stained with H&E (Fig. 7A). No tissue lesions was observed. Additionally, no significant difference was found the various blood routine markers in the serum, including alanine transferase (ALT), aspartate transferase (AST), creatinine (Crea), creatine kinase (CK), total bilirubin (TBIL), total cholesterol (T-CHO), triglyceride (TG), or urea (Fig. 7B).

## Conclusion

In this study, BBR and MAG, as a pair of natural compounds were, found to self-assemble into nanoassembly BM without any carrier. Spectral characterization, including UV, XRD, FTIR, and NMR, confirmed this self-assembly and elucidated the underlying reaction mechanism of BM. We also demonstrated the promotion of BM on BBR in vivo processes from the perspectives of small molecule content and supramolecular distribution. Compared to separate administration of BBR or MAG, BM exhibited specific advantages in regulating inflammatory cytokines, restoring tight junction proteins, and regulating the gut microbiota. Furthermore, no safety issues were observed after oral administration of BM. We provided evidence that BBR improves its bioavailability, colon accumulation and anti-inflammatory effects through its assembly with MAG, while there is still a lack of further research on the molecular and cellular transport mechanisms of BM. This discovery offers new strategies for the treatment of UC and revealed the mechanisms of this popular herbal pair. Such an approach has yet to be explored for other natural product combinations to identify stronger drug treatments and address other gastrointestinal disorders. As the self-assembly of phytochemical-based molecules becomes increasingly popular as a strategy to develop bioactive nanomaterials, MD computation could be an effective tool for structural modification or screening of compound series [36]. We believe that correlating experimental parameters with the molecular fingerprints of natural products will advance the design and prediction of self-assembly systems.

## Electronic supplementary material

Below is the link to the electronic supplementary material.

Supplementary Material 1

## Data Availability

No datasets were generated or analysed during the current study.
