# Supplementary material for: Berberine and magnolol exert cooperative effects on ulcerative colitis in mice by self-assembling into carrier-free nanostructures
Source: J Nanobiotechnology. 2024 Sep 4;22:538. doi: 10.1186/s12951-024-02804-x (PMC11373475; doi:10.1186/s12951-024-02804-x)
Supplement: Supplementary file 1 — Supplementary Material 1 [file 12951_2024_2804_MOESM1_ESM.docx]

**SUPPLEMENTARY MATERIAL**

**Berberine and magnolol exert a cooperative effect on ulcerative colitis in mice by self-assembling into carrier-free nanostructures**

Yida Xu^a,b^, Zhejie Chen^c^, Wei Hao^a,b^, Zhengming Yang^a,b^, Mohamed Farag^d^, Chi Teng Vong^a,b^,Yitao Wang^a,b,*^, Shengpeng Wang^a,b,*^

**Experimental section:**

*UV spectra analysis:* The BBR, MAG, and BM powder were dissolved in dilute NaOH solution (pH 12) at 0.02 mmol/mL. A full scan of 200-600 nm wavelength was used with a step of 1 nm, and the NaOH was subtracted as a blank.

*NMR analysis:* 5 mg BBR, MAG, and BM were dissolved in nuclear magnetic tube with 0.6 ml DMSO-d6, respectively. The chemical shift (*δ*) is measured in ppm and the coupling constant (J) is measured in Bruker (Ultrashield 400 Plus, 400 MHz). The acquisition mode of NOESY is DQD. The size of fid of F1 and F2 is 2048 and 256, respectively.

*MS conditions for Pharmacokinetic studies:*

Column: Agilent ZORBAX SB-C18, 5 *µ*m, 4.6 × 150 mm

Mobile phase: A: methanol/B: 0.1% formic acid in water (65% A/35% B, v/v)

Flow Rate: 1 mL/min

Injection Volume: 5.00 *µ*L

Column temperature: 35℃

The MS method is as follows:

Ion source: Electrospray Ionization (ESI)

Ionization mode: Positive ion mode

Detection mode: Multiple reaction monitoring (MRM)

Ion Spray Voltage: 5500 V

Turbo Ion Spray Temperature: 550℃

Curtain Gas Type: Nitrogen Setting: 20 psi

Nebulizing Gas (Gas1): Nitrogen Setting: 30 psi

Auxiliary Gas (Gas 2): Nitrogen Setting: 30 psi

Data acquisition time: 3.0 min

**Table S1.** Analytical content in pharmacokinetic studies

|  | Compound | Monitored ion transitions (MRM) | Dwell time (ms) | CXP  (volts) | CE (volts) | Retention time  (min) |
| --- | --- | --- | --- | --- | --- | --- |
| Analyte | Berberine | 337.200→321.200 | 200.00 | 19.00 | 43.00 | 2.000 |
| Internal standard | Berberine -d_6_ | 343.200→325.100 | 200.00 | 16.00 | 34.00 | 2.012 |

*Immunofluorescence Staining*

For IF staining, frozen colon sections were fixed in 4% paraformaldehyde, permeabilized with 0.1% Triton X-100, and blocked for non-specific binding using 1% BSA in PBS for 1 h. The sections were then incubated overnight at 4°C with primary antibodies against *β*-catenin, apo-BrdU, ZO-1, and occludin, appropriately diluted. Following incubation, the confocal dishes and slides were washed with PBS and incubated overnight at 4°C with secondary antibodies, including Alexa Fluor 488 and/or Sulfo-Cyanine 3, along with DAPI dihydrochloride for nuclear staining.

*Bioinformatics analysis:*

Raw FASTQ files were de-multiplexed using an in-house perl script, and then quality-filtered by fastp version 0.19.6 [1] and merged by FLASH version 1.2.7 [2] with the following criteria:

1. The reads were truncated at any site receiving an average quality score of < 20 over a 50 bp sliding window, and the truncated reads shorter than 50 bp were discarded, reads containing ambiguous characters were also discarded; (ii) Only overlapping sequences longer than 10 bp were assembled according to their overlapped sequence. The maximum mismatch ratio of overlap region is 0.2. Reads that could not be assembled were discarded; (iii) Samples were distinguished according to the barcode and primers, and the sequence direction was adjusted, exact barcode matching, 2 nucleotide mismatch in primer matching. Then the optimized sequences were clustered into operational taxonomic units (OTUs) using UPARSE 7.1 [3] with 97% sequence similarity level. The most abundant sequence for each OTU was selected as a representative sequence.

The taxonomy of each OTU representative sequence was analyzed by RDP Classifier version 2.2 against the 16S rRNA gene database (eg. Silva v138) using confidence threshold of 0.7. The metagenomic function was predicted by PICRUSt2 (Phylogenetic Investigation of Communities by Reconstruction of Unobserved States) based on OTU representative sequences. PICRUSt2 is a software containing a series of tools as follows: HMMER was used to aligns OTU representative sequences with reference sequences. EPA-NG and Gappa were used to put OTU representative sequences into a reference tree. The castor was used to normalize the 16S gene copies.

statistical method:

*Taxonomic analysis*

The sequences were clustered into OTUs at 97% sequence identity using the UPARSE (V 7.0.1090) [3], resulting in 1,234 OTUs. Representative sequences from each OTU were were taxonomically annotated using RDP Classifier [4] with a confidence cut-off of 70%, using the SILVA v132 database. Sequences identified as mitochondria and chloroplast were removed from the dataset. To avoid bias in sampling effort, samples were rarefied to 30,000 sequences, resulting in 1,134 OTUs overall, which were used in subsequent comparative analyses.

Alpha diversity analysis and dilution curve

Rarefaction curves construction as well as alpha diversity indices were calculated on rarified data set (30,000 sequences for bacteria) using the Mothur (v1.30.2). Alpha diversity indices were compared with Kruskal Wallis test using the stats package in R (v3.3.1).

*Venn diagram analysis*

The Venn diagram constructed using jven（http://jvenn.toulouse.inra.fr/app/index.html）with shared and unique OTUs was used to depict the similarity and difference between the two communities.

*Community Bar Chart*

Stacked bar plot conducted by R (v3.3.1) was used to identify the most abundance bacterial communities both on phylum and genus levels. Bar charts indicate the relative proportion of each taxon (phylum and genus levels), size of bar chart indicates relative abundance. Taxonomic groups with a relative abundance of under 1% in all samples were combined into others.

*PCOA/NMDS analysis*

The similarity among the microbial communities was determined by PCoA (Principal coordinate analysis) / NMDS (Non-metric multidimensional scaling) based on Bray-Curtis distance using vegan package in R (v 3.3.1).

*Lefse analysis*

The linear discriminant analysis (LDA) effect size (LEfSe) [5] (http://huttenhower.sph.harvard.edu/LEfSe) was performed to identify differentially abundant taxa (phylum to genera) among the different samples (LDA score > 4, P < 0.05). This method firstly uses the nonparametric factorial Kruskal-Wallis rank-sum test to detect features with significant differential abundance and then uses linear discriminant analysis (LDA) to calculate the effect size of each feature.

**Supporting results**


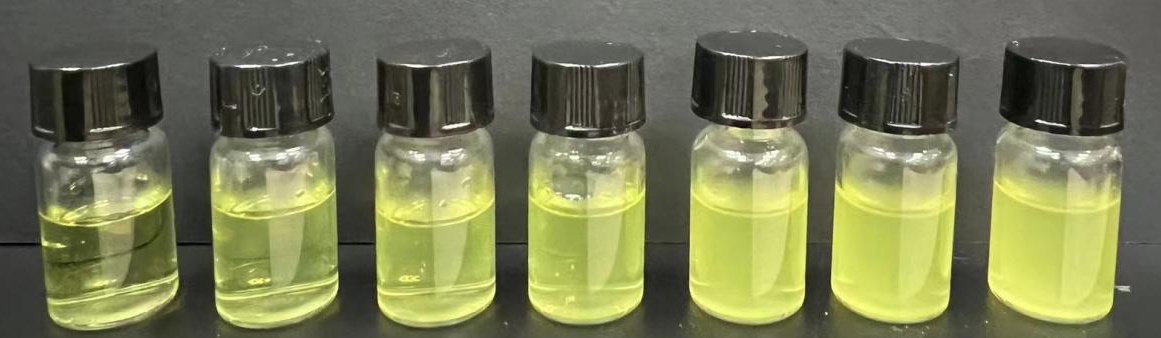


**Fig. S1**. The image of different ratio of BM selfassemblies. The molar ratio of BBR and MAG From left to right are 4:1, 3:1, 2:1, 1:1, 1:2, 1:3, 1:4 (BBR = 10 mmol/L), respectively.

**
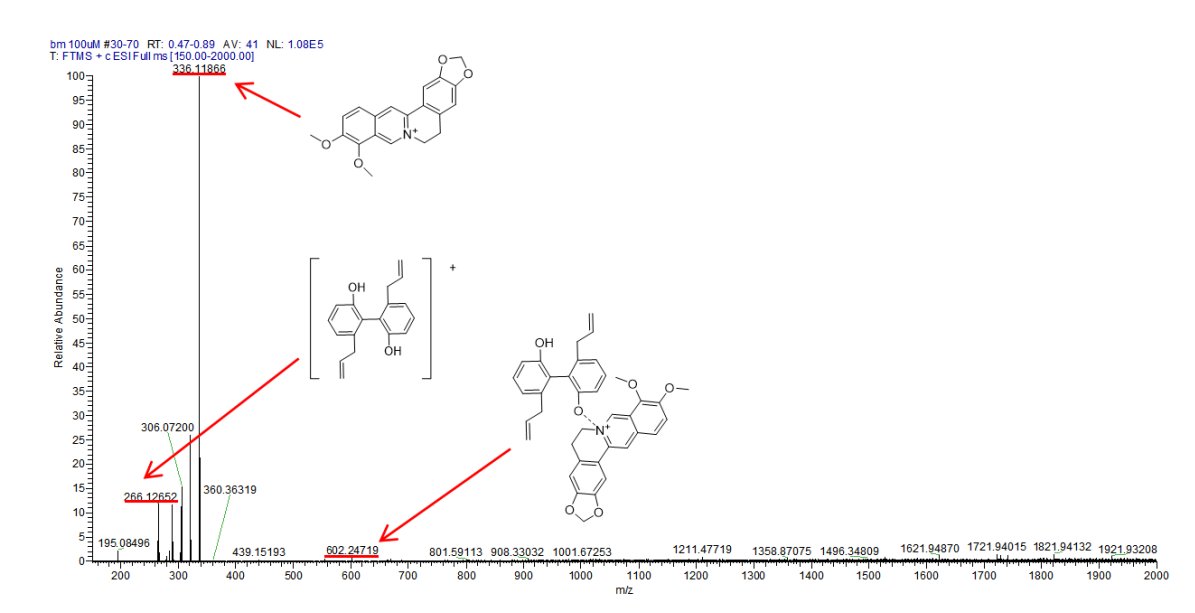
**

**Fig. S2**. MS spectrum of BM.

**
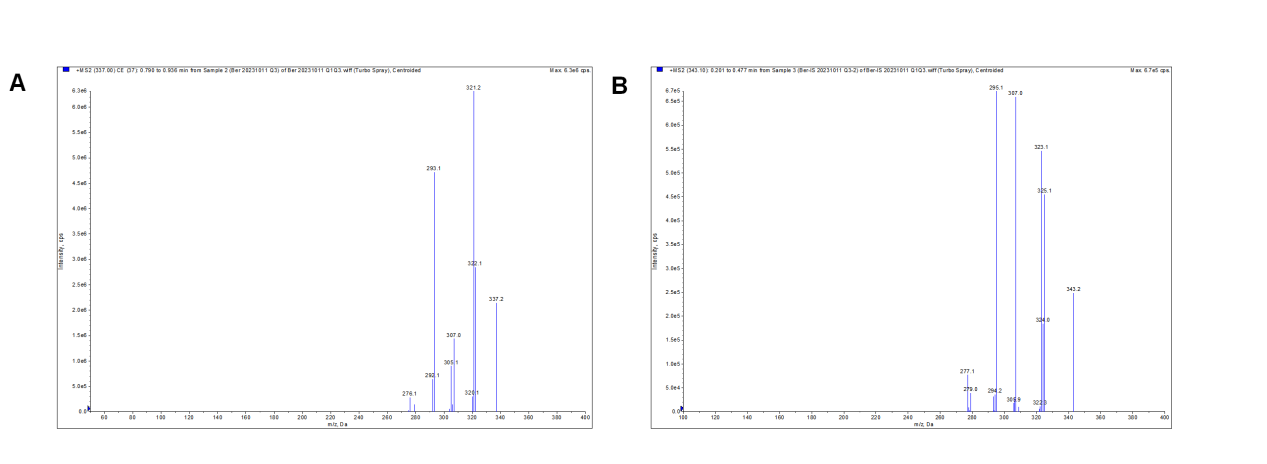
**

**Fig. S3.** Product ion full scan mass spectrum of (A) berberine [M+H]^+^ and (B) berberine-d6 [M+H]^+^

**
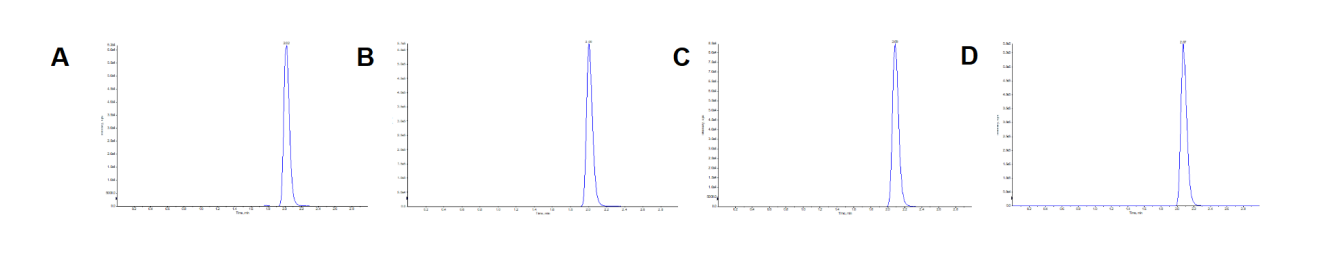
**

**Fig. S4**. Representative chromatogram (A) Chromatogram of plasma sample (BBR) (B) chromatogram of plasma sample (BBR-d6) (C) Chromatogram of colon sample (BBR) (D) Chromatogram of colon sample (BBR-d6)

**Table S2.** Analytical content in pharmacokinetic studies

| Slope | Intercept | r^2^ | Weight factor | Standard curve range (ng/mL) |
| --- | --- | --- | --- | --- |
| 0.00261 | 0.002770 | 0.9954 | 1/x^2^ | 1.00 ~ 1000.00 |

**Table S3.** Standard curve parameters for BBR determination

| Slope | Intercept | r^2^ | Weight factor | Standard curve range (*μ*g/mL) |
| --- | --- | --- | --- | --- |
| 97.5 | 5200 | 0.99732 | x | 50.00 ~ 5000.00 |

**Table 6:** Accuracy and precision of pharmacokinetic testing

| Batch number | QC-L | | QC-M | | QC-H | |
| --- | --- | --- | --- | --- | --- | --- |
|  | 2.50 ng/mL | | 100.00 ng/mL | | 800.00 ng/mL | |
|  | Measured concentration | Accuracy deviation (%) | Measured concentration | Accuracy deviation (%) | Measured concentration | Accuracy deviation (%) |
|  | (ng/mL) |  | (ng/mL) |  | (ng/mL) |  |
| 1 | 2.58 | 103.25 | 103.32 | 103.32 | 750.64 | 93.83 |
|  | 2.45 | 98.15 | 107.11 | 107.11 | 742.28 | 92.79 |
|  | 2.5 | 100.07 | 106.26 | 106.26 | 750.48 | 93.81 |
| Intra batch average | 2.51 | NA | 105.56 | NA | 747.8 | NA |
| Intra batch SD | 0.07 |  | 1.99 |  | 4.78 |  |
| Intra batch RSD (%) | 2.61 |  | 1.88 |  | 0.64 |  |
| Intra batch Average accuracy deviation (%) | 0.4 |  | 5.56 |  | -6.52 |  |
| n | 3 |  | 3 |  | 3 |  |
| 2 | 2.51 | 100.21 | 100.54 | 100.54 | 705.96 | 88.24 |
|  | 2.48 | 99.33 | 104.44 | 104.44 | 701.7 | 87.71 |
|  | 2.47 | 98.78 | 98.94 | 98.94 | 698.02 | 87.25 |
| Intra batch average | 2.49 | NA | 101.31 | NA | 701.89 | NA |
| Intra batch SD | 0.02 |  | 2.83 |  | 3.97 |  |
| Intra batch RSD (%) | 0.84 |  | 2.79 |  | 0.57 |  |
| Intra batch Average accuracy deviation (%) | -0.53 |  | 1.31 |  | -12.26 |  |
| n | 3 |  | 3 |  | 3 |  |
| 3 | 2.53 | 101.07 | 107.19 | 107.19 | 756.55 | 94.57 |
|  | 2.55 | 101.96 | 104.13 | 104.13 | 710.64 | 88.83 |
|  | 2.39 | 95.79 | 108.29 | 108.29 | 762.74 | 95.34 |
| Intra batch average | 2.49 | NA | 106.54 | NA | 743.31 | NA |
| Intra batch SD | 0.09 |  | 2.16 |  | 28.46 |  |
| Intra batch RSD (%) | 3.5 |  | 2.02 |  | 3.83 |  |
| Intra batch Average accuracy deviation (%) | -0.4 |  | 6.54 |  | -7.09 |  |
| n | 3 |  | 3 |  | 3 |  |
| Inter batch mean | 2.5 | NA | 104.47 | NA | 731 | NA |
| Inter batch SD | 0.01 |  | 2.78 |  | 25.31 |  |
| Inter batch RSD (%) | 0.51 |  | 2.66 |  | 3.46 |  |
| Inter batch Average accuracy deviation (%) | -0.18 |  | 4.47 |  | -8.62 |  |
| n | 9 |  | 9 |  | 9 |  |

**Table S5:** Scoring system for calculating disease activity index (DAI)

| Score | Body Weight Loss (%) | Stool Consistency | Rectal Bleeding |
| --- | --- | --- | --- |
| 0 | 0 | Normal | Normal |
| 1 | 1-5 |  |  |
| 2 | 5-10 | Loose stools |  |
| 3 | 10-20 |  |  |
| 4 | > 20 | Diarrhea | Gross bleeding |

**References:**

1. Chen SF, Zhou YQ, Chen YR, Gu J: fastp: an ultra-fast all-in-one FASTQ preprocessor. *Bioinformatics* 2018, 34(17):884-890.

2. Magoc T, Salzberg SL: FLASH: fast length adjustment of short reads to improve genome assemblies. *Bioinformatics* 2011, 27(21):2957-2963.

3. Edgar RC: UPARSE: highly accurate OTU sequences from microbial amplicon reads. *Nature Methods* 2013, 10(10):996-+.

4. Wang Q, Garrity GM, Tiedje JM, Cole JR: Naive Bayesian classifier for rapid assignment of rRNA sequences into the new bacterial taxonomy. *Applied and Environmental Microbiology* 2007, 73(16):5261-5267.

5. Segata N, Izard J, Waldron L, Gevers D, Miropolsky L, Garrett WS, Huttenhower C: Metagenomic biomarker discovery and explanation. *Genome Biology* 2011, 12(6).
